# Postoperative adjuvant therapy following radical resection for intrahepatic cholangiocarcinoma: A multicenter retrospective study

**DOI:** 10.1002/cam4.2925

**Published:** 2020-02-19

**Authors:** Lei Wang, Manjun Deng, Qiao Ke, Jianying Lou, Shuguo Zheng, Xinyu Bi, Jianming Wang, Wei Guo, Fuyu Li, Jian Wang, Yamin Zheng, Jingdong Li, Shi Cheng, Weiping Zhou, Yongyi Zeng

**Affiliations:** ^1^ Department of Radiation Oncology Mengchao Hepatobiliary Hospital of Fujian Medical University Fuzhou China; ^2^ Department of Hepatobiliary Surgery Mengchao Hepatobiliary Hospital of Fujian Medical University Fuzhou China; ^3^ Department of Hepatobiliary Surgery The Second Hospital Affiliated to Zhejiang University Hangzhou China; ^4^ Department of Hepatobiliary Surgery The Southwest Hospital Affiliated to the Army Medical University Chongqing China; ^5^ Department of Hepatobiliary Surgery Cancer Hospital Chinese Academy of Medical Sciences Beijing China; ^6^ Department of Hepatobiliary Surgery Tongji Hospital Affiliated to Tongji Medical College Huazhong University of Science & Technology Wuhan Hubei China; ^7^ Department of Hepatobiliary Surgery Beijing Friendship Hospital Affiliated to Capital Medical University Beijing China; ^8^ Department of Hepatobiliary Surgery The West China Hospital of Sichuan University Chengdu China; ^9^ Department of Hepatobiliary Surgery Renji Hospital Affiliated to Shanghai Jiaotong University Shanghai China; ^10^ Department of Hepatobiliary Surgery Xuanwu Hospital Affiliated to Capital Medical University Beijing China; ^11^ Department of Hepatobiliary Surgery The Affiliated Hospital of Chuanbei Medical University Nanchong China; ^12^ Department of Hepatobiliary Surgery Tiantan Hospital Affiliated to Capital Medical University Beijing China; ^13^ Department of Hepatobiliary Surgery Ⅲ Eastern Hepatobiliary Surgery Hospital Secondary Military Medical University Shanghai China

**Keywords:** adjuvant therapy, disease‐free survival, intrahepatic cholangiocarcinoma, overall survival, propensity score matching

## Abstract

**Background and Aims:**

The prognosis of intrahepatic cholangiocarcinoma (ICC) after radical resection is far from satisfactory; however, the clinical value of adjuvant therapy (AT) remains controversial. This multicenter study aimed to evaluate the clinical value of AT and identify potential patients who would be benefited from AT.

**Methods:**

Data from ICC patients who underwent radical resection were retrospectively collected from 12 hepatobiliary centers in China between December 2012 and December 2015. Patients were divided into AT and non‐AT groups based on whether AT was administered or not. Overall survival (OS) and disease‐free survival (DFS) were analyzed using the Kaplan‐Meier method before and after 1:2 propensity score matching (PSM). Subgroup analyses were conducted based on the established staging systems.

**Results:**

A total of 412 patients were enrolled in this study, and 77 patients (18.9%) received AT, including 32 (7.8%) patients who received transarterial chemoembolization (TACE), 21 (5.1%) patients who received chemotherapy, 10 (2.4%) patients who received radiotherapy, and 14 (3.4%) patients who received adjuvant chemoradiotherapy. The median OS and DFS were both longer in the AT group than in the non‐AT group (43.0 months vs 21.0 months, *P* = .015; 16.0 months vs 11.0 months, *P* = .045, respectively), and the advantage of AT was confirmed for both the OS and DFS (*P* = .023; *P* = .046, respectively) after 1:2 PSM. Furthermore, based on the established nomogram, only “middle‐risk” patients receiving AT cherished a longer median OS (43.0 months vs 20.0 months, *P* = .033). In subgroup analyses that were stratified by different AT strategies, patients receiving postoperative chemotherapy had a longer median OS (37.0 months vs 21.0 months, *P* = .039), while patients receiving postoperative TACE had a longer median DFS (50.0 months vs 11.0 months, *P* = .007).

**Conclusion:**

With the current data, we conclude that AT benefits ICC patients following radical resection, especially those “middle‐risk” patients, as evaluated by the established nomogram. However, exactly which patients are the most suitable for AT requires further study and validation.

## Graphical abstract

The prognosis of intrahepatic cholangiocarcinoma after radical resection is far from satisfactory, but the clinical value of postoperative adjuvant therapy remains controversial. This study was to evaluate the clinical value of adjuvant therapy and identify the selected patients benefited from adjuvant therapy in a multicenter study.

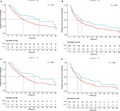

## 1 INTRODUCTION

Intrahepatic cholangiocarcinoma (ICC) is the second most common primary liver cancer after hepatocellular carcinoma. The incidence of ICC is increasing worldwide, with an average 2.3% annual increase.^1^, ^2^ The 5‐year survival rate for patients with ICC is less than 10%,^3^, ^4^ partly because approximately 80% of patients are diagnosed at a late stage when surgery is no longer a treatment option.^5^, ^6^ Currently, radical resection is the only potentially curative strategy for patients with ICC,^5^, ^6^ but the 5‐year survival rate even after radical resection is far from satisfactory. Hence, adjuvant therapy (AT) is badly warranted to improve the prognosis of ICC.

Recently, AT, such as transarterial chemoembolization (TACE), chemotherapy, radiotherapy, and comprehensive treatment, has been conducted prevalently worldwide to improve ICC patient prognosis after radical resection.^7^, ^8^, ^9^, ^10^, ^11^, ^12^, ^13^, ^14^ However, there is still controversy over whether ICC patients can benefit from AT after radical resection.^15^, ^16^, ^17^ The key is to identify those ICC patients who would benefit from AT, and exactly what form of AT is the most beneficial. Randomized controlled trials are hard to conduct due to the rare morbidity of ICC. Therefore, we conducted this retrospective multicenter study to identify those patients who benefited from AT.

## 2 MATERIAL AND METHODS

### 2.1 Patient selection

This study was conducted according to the ethical guidelines of the 1975 Declaration of Helsinki and was approved by all 12 participating centers, including Mengchao hepatobiliary hospital, Eastern hepatobiliary surgery hospital, Affiliated Cancer Hospital of Chinese Academy of Medical Sciences, Tongji Hospital, Beijing Friendship Hospital, Xuanwu Hospital, Tiantan Hospital, affiliated Hospital of Chuanbei Medical University, Renji Hospital, West China Hospital, Southwest Hospital, and Second Hospital of Zhejiang University. Data, including baseline characteristics, operation parameters, and tumor characteristics, were collected via an electric case report form between December 2012 and December 2015.

### 2.2 Eligibility

Patients were enrolled into this study if they: (a) had a confirmed histopathological diagnosis of ICC; (b) underwent an R0 resection with or without lymph node dissection (LND) and experienced no recurrence within 2 months of surgery; and (c) received postoperative AT, such as TACE, chemotherapy, radiotherapy, and comprehensive treatment, or not. Patients who met any of the following criteria were excluded from the study: (a) incomplete clinical data, (b) preoperative obstructive jaundice, (c) extrahepatic metastasis, (d) a positive margin, (e) mortality within 1 month of surgery, and (f) AT after recurrence.

### 2.3 Interventions

A R0 resection was achieved by hepatectomy, with or without LND, albeit with slight procedural differences among different centers.

Those patients who underwent an R0 resection with no recurrence within 2 months of surgery were classed as having undergone a radical resection.

Following an assessment by a multidisciplinary team, patients underwent AT, such as TACE, chemotherapy, radiotherapy, and chemoradiotherapy, after radical resection, with the aim of reducing the risk of recurrence and improving prognosis.

One or two courses of TACE were conducted between 3 weeks and 2 months after resection. The most commonly used chemotherapeutic agents were 5‐fluorouracil (500 mg), epirubicin (20 mg), and hydroxycamptothecin (10 mg) with an emulsion of lipiodol (5‐10 mL). Adjuvant chemotherapy was often conducted within 1‐2 months following resection, and 4‐6 courses of fluoropyrimidine‐ or gemcitabine‐based chemotherapy regimens were used most frequently. Adjuvant radiotherapy was performed within 4‐8 weeks following resection, and intensity‐modulated radiation therapy with a total dose of 45‐50 Gy at 1.8‐2.0 Gy/fractions was the preferred option. Where a sequential chemoradiotherapy regimen was used, chemotherapy was often conducted after radiotherapy. Radiotherapy was targeted at the tumor bed with or without potential lymphatic drainage region.

### 2.4 Follow‐up and definition of endpoints

All patients were periodically followed‐up every 2‐3 months in the first 2 years after resection, and then once every 6 months. Routine follow‐up tests included liver function tests, serum levels of carbohydrate antigen 199 (CA19‐9) and carcinoembryonic antigen (CEA), and an abdominal ultrasound. Contrast‐enhanced computed tomography or magnetic resonance imaging was only warranted once recurrence was clinically suspected. Recurrence or metastasis was defined as the appearance of new lesions with the same radiologic characteristics of ICC, and further treatment was started immediately whenever recurrence was confirmed.

The primary endpoint of this study was overall survival (OS), and the secondary endpoint was disease‐free survival (DFS). OS was calculated from the date of resection to either the date of death or the latest follow‐up. DFS was defined as from the time of resection to the time of recurrence (intrahepatic or extrahepatic) or the date of the latest follow‐up.

### 2.5 Propensity score matching

Propensity score matching (PSM) was adopted to minimize selection bias, and the propensity score was determined using potential confounding factors. Patients were matched using a 1:2 ratio and the nearest neighbor method, with a caliper of 0.2.

### 2.6 Statistics

Continuous variables were all re‐defined as categorical variables, hence, they were all evaluated using a Chi‐square test or Fisher's exact test between the two groups. Survival curves were determined by the Kaplan‐Meier method, both before and after PSM, and median OS and DFS were evaluated for the AT and non‐AT groups with hazard ratio (HR) and confidence interval (CI) 95%. A univariate analysis was used to identify prognostic factors of OS and DFS in ICC patients following radical resection, both before and after PSM. A multivariable Cox proportional hazards model of those prognostic factors with a *P* < .2 was then executed to determine potential independent risk factors.

A subgroup analysis of the following variables was performed using the Kaplan‐Meier method to determine the OS of ICC patients receiving AT in the whole cohort; gender (female vs male), age (≤50 years vs >50 years), hepatitis (no vs yes), CA19‐9 (≤37 U/mL vs >37 U/mL), Child‐Pugh (grade A vs grade B), intraoperative blood loss (≤400 mL vs >400 mL), tumor size (≤3 cm vs 3‐5 cm vs >10 cm), tumor number (single vs multiple), surgical margin (<1 cm vs ≥1 cm), differentiation (well‐moderate vs poor), satellite (no vs yes), microvascular invasion (MVI) (no vs yes), and lymph node metastasis (LNM) (no vs yes). A forest plot of the subgroup analysis was depicted with each estimated HR and 95% CI.

Patients from the AT and non‐AT groups were then subdivided into groups according to the 8th American Joint Committee on Cancer (AJCC) staging system and the established nomogram.^5^, ^11^ The median OS for each AT and non‐AT subgroups was then calculated using the Kaplan‐Meier method.

In addition, a subgroup analysis of different AT strategies was evaluated between the AT and non‐AT groups. Data analysis was conducted using Rstudio 3.6.1 software that included packages of “table1,” “MatchIt,” “survminer,” “survival,” “plyr,” and “forestplot.” A two‐tailed *P* < .05 was considered statistically significant.

## 3 RESULTS

### 3.1 Baseline characteristics

**Figure 1:**
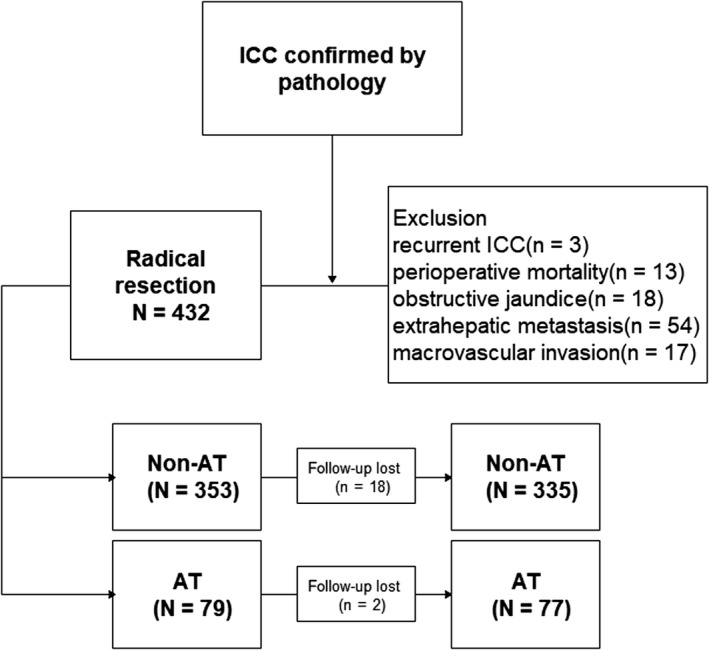
Flow chart of patients' enrollment

Initially, 537 patients with ICC underwent resection, but 105 patients were excluded from the study for the following reasons; 18 patients (3.4%) for preoperative obstructive jaundice, three (0.5%) for ICC recurrence, 13 (2.4%) died within 1 month of resection, 54 (10.1%) for extrahepatic metastasis, and 17 (3.2%) for macrovascular invasion. Finally, 432 patients were enrolled in this study. During the median follow‐up of 22 months, 20 patients (4.6%) were not followed‐up, leaving 412 patients whose details were analyzed (Figure 1).

The baseline characteristics of the 412 patients analyzed in the study are shown in Table 1. The median patient age was 56 years, and 260 patients (63.1%) were male. The median size of the resected tumor was 6.5 cm, and 296 patients (71.8%) had a single tumor. In total, 109 patients (26.5%) underwent LND, among whom 53 patients (48.6%) were postoperatively confirmed as having LNM.

**Table 1:** Clinicopathological characteristics before and after PSM

Characteristics	Before PSM			After PSM		
	Non‐AT (n = 335)	AT (n = 77)	*P*‐value	Non‐AT (n = 154)	AT (n = 77)	*P*‐value
Gender						
Female	127 (37.9%)	25 (32.5%)	.446	63 (40.9%)	25 (32.5%)	.271
Male	208 (62.1%)	52 (67.5%)		91 (59.1%)	52 (67.5%)	
Age						
≤50	94 (28.1%)	30 (39.0%)	.081	55 (35.7%)	30 (39.0%)	.736
>50	241 (71.9%)	47 (61.0%)		99 (64.3%)	47 (61.0%)	
HBV						
No	217 (64.8%)	49 (63.6%)	.955	98 (63.6%)	49 (63.6%)	1
Yes	118 (35.20%)	28 (36.4%)		56 (36.4%)	28 (36.4%)	
CA19‐9 (U/mL)						
≤37	224 (66.9%)	49 (63.6%)	.684	99 (64.3%)	49 (63.6%)	1
>37	111 (33.1%)	28 (36.4%)		55 (35.7%)	28 (36.4%)	
Child‐Pugh						
A	186 (55.5%)	52 (67.5%)	.073	93 (60.4%)	52 (67.5%)	.361
B	149 (44.5%)	25 (32.5%)		61 (39.6%)	25 (32.5%)	
Blood loss (mL)						
≤400	242 (72.2%)	47 (61.0%)	.072	93 (60.4%)	47 (61.0%)	1
>400	93 (27.8%)	30 (39.0%)		61 (39.6%)	30 (39.0%)	
Margin						
Wide	114 (34.0%)	35 (45.5%)	.080	64 (41.6%)	35 (45.5%)	.672
Narrow	221 (66.0%)	42 (54.5%)		90 (58.4%)	42 (54.5%)	
Differentiation						
Well and moderate	265 (79.1%)	59 (76.6%)	.745	123 (79.9%)	59 (76.6%)	.690
Poor	70 (20.9%)	18 (23.4%)		31 (20.1%)	18 (23.4%)	
Tumor size (cm)						
≤3	35 (10.4%)	10 (13.0%)	.064	28 (18.2%)	10 (13.0%)	.344
3 ~ 5	83 (24.8%)	28 (36.4%)		43 (27.9%)	28 (36.4%)	
>5	217 (64.8%	39 (50.6%)		83 (53.9%)	39 (50.6%)	
Tumor number						
Single	237 (70.7%	59 (76.6%)	.372	120 (77.9%)	59 (76.6%)	.956
Multiple	98 (29.3%)	18 (23.4%)		34 (22.1%)	18 (23.4%)	
Satellite						
No	246 (73.4%	62 (80.5%)	.252	122 (79.2%)	62 (80.5%)	.954
Yes	89 (26.6%)	15 (19.5%)		32 (20.8%)	15 (19.5%)	
MVI						
No	302 (90.1%	70 (90.9%)	1	146 (94.8%)	70 (90.9%)	.396
Yes	33 (9.9%)	7 (9.1%)		8 (5.2%)	7 (9.1%)	
LNM						
No	295 (88.1%)	61 (79.2%)	.095	125 (81.2%)	61 (79.2%)	.860
Yes	40 (11.9%)	16 (20.8%)		29 (18.8%)	16 (20.8%)	
AJCC^a^						
I	198 (59.1%)	44 (57.1%)	.095	98 (63.6%)	44 (57.1%)	.604
II	97 (29.0%)	17 (22.1%)		27 (17.5%)	17 (22.1%)	
III	40 (11.9%)	16 (20.8%)		29 (18.8%)	16 (20.8%)	
Nomogram						
Mean (SD)	84.8 (209)	70.4 (63.9)	.290	99.4 (293)	70.4 (63.9)	.243

Abbreviations: AJCC, the American Joint Committee on Cancer; AT, adjuvant therapy; HBV, hepatitis B virus; LNM, lymph node metastasis; MVI, microvascular invasion; PSM, propensity score matching; SD, standard deviation.

a According to the 8th edition AJCC staging guidelines.^5^

Of note, the clinicopathological characteristics of the AT and non‐AT groups were almost statistically comparable (Table 1), but, nonetheless, we conducted 1:2 PSM to decrease the potential for confounding factors (Table 2).

**Table 2:** Univariate and multivariate analysis of overall survival and disease‐free survival for patients with intrahepatic cholangiocarcinoma in a whole cohort

Characteristics	OS				DFS			
	Univariate		Multivariate		Univariate		Multivariate	
	HR (95CI)	*P‐*value	HR (95CI)	*P‐*value	HR (95CI)	*P‐*value	HR (95CI)	*P‐*value
Gender (female as ref)								
Male	1.36 (1.04‐1.77)	.023	1.48 (1.13‐1.93)	.004	1.32 (1.04‐1.66)	.02	1.42 (1.12‐1.79)	.004
Age (≤50 y as ref)								
>50 y	0.96 (0.74‐1.25)	.772			0.91 (0.72‐1.15)	.435		
HBV (no as ref)								
Yes	0.90 (0.70‐1.17)	.439			0.84 (0.66‐1.06)	.139	0.95 (0.74‐1.21)	.672
CA19‐9 (≤37 U/mL as ref)								
>37 U/mL	1.28 (0.99‐1.66)	.062	1.38 (1.03‐1.85)	.030	1.13 (0.89‐1.43)	.310		
Child‐Pugh (A as ref)								
B	1.17 (0.92‐1.5)	.207			1.17 (0.94‐1.46)	.159		
Blood loss (≤400 mL as ref)								
>400 mL	0.98 (0.74‐1.29)	.878			0.96 (0.75‐1.23)	.750		
Margin (wide ≥1 cm as ref)								
Narrow	1.44 (1.1‐1.88)	.008	1.35 (1.00‐1.83)	.053	1.42 (1.12‐1.80)	.004	1.14 (0.87‐1.49)	.333
Differentiation (well and moderate as ref)								
Poor	0.91 (0.67‐1.23)	.525			0.78 (0.59‐1.03)	.085	0.89 (0.66‐1.21)	.459
Tumor size (≤3 cm as ref)								
3 ~ 5 cm	1.60 (0.93‐2.73)	.089	1.54 (0.89‐2.64)	.122	1.29 (0.83‐2.00)	.250	1.24 (0.79‐1.93)	.344
>5 cm	2.61 (1.59‐4.31)	<.001	2.27 (1.37‐3.77)	.002	2.03 (1.36‐3.04)	.001	1.80 (1.18‐2.74)	.006
Tumor number (single as ref)								
Multiple	1.84 (1.41‐2.4)	<.001	0.97 (0.59‐1.58)	.898	1.84 (1.44‐2.35)	<.001	1.12 (0.72‐1.72)	.614
Satellite (no as ref)								
Yes	2.20 (1.68‐2.88)	<.001	1.86 (1.14‐3.03)	.013	2.14 (1.67‐2.74)	<.001	1.65 (1.06‐2.56)	.026
MVI (no as ref)								
Yes	1.71 (1.16‐2.52)	.007	1.49 (0.99‐2.23)	.054	1.36 (0.94‐1.96)	.104	1.29 (0.88‐1.88)	.197
LNM (no as ref)								
Yes	2.00 (1.42‐2.83)	.001	1.82 (1.25‐2.63)	.002	1.60 (1.17‐2.2)	.003	1.61 (1.15‐2.25)	.005
Postoperative AT (no as ref)								
Yes	0.63 (0.44‐0.92)	.015	0.70 (0.48‐1.03)	.068	0.73 (0.53‐0.99)	.045	0.80 (0.58‐1.11)	.177

Abbreviations: AT, adjuvant therapy; CI, confidence interval; DFS, disease‐free survival; HBV, hepatitis B virus; HR, hazard ratio; LNM, lymph node metastasis; MVI, microvascular invasion; OS, overall survival.

### 3.2 Long‐term outcomes

**Figure 2:**
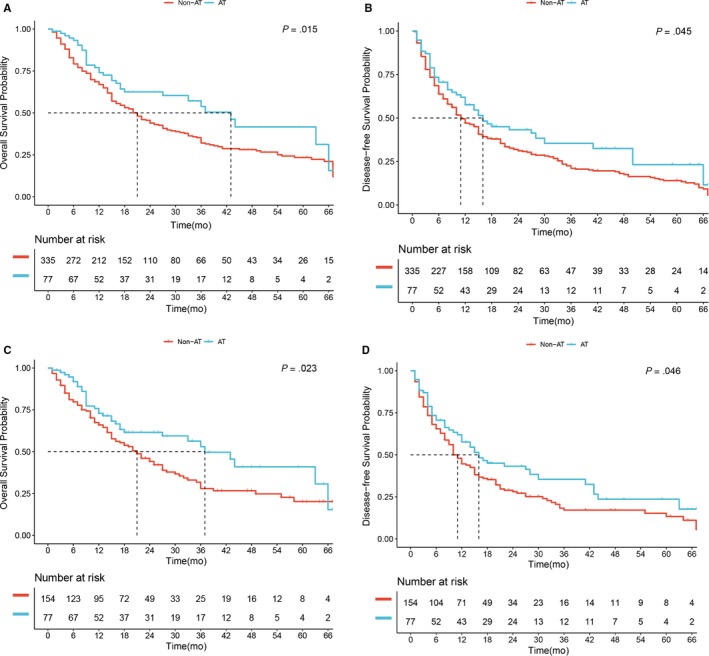
Overall survival (OS) (A) and disease‐free survival (DFS) (B) of patients receiving adjuvant therapy (AT) or not in a whole cohort. OS (C) and DFS (D) of patients receiving AT or not after 1:2 propensity score matching

The median OS was longer in the AT than in the non‐AT group (43.0 months vs 21.0 months, *P* = .015; Figure 2A). The 1‐, 3‐, and 5‐year OS rates were also significantly higher in the AT group than in the non‐AT group (74% vs 63%, 50% vs 31%, 43% vs 24%, all *P* < .05, respectively). The median DFS time was longer in the AT than in the non‐AT group (16.0 months vs 11.0 months, *P* = .045; Figure 2B). The 1‐, 3‐, and 5‐year DFS rates were also significantly higher in the AT group than in the non‐AT group (56% vs 46%, 36% vs 20%, 24% vs 14%, all *P* < .05, respectively).

After 1:2 PSM, the median OS time was still longer in the AT than in the non‐AT group (37.0 months vs 21.0 months, *P* = .023; Figure 2C), and the 1‐, 3‐, and 5‐year OS rates were still significantly higher in the AT group than in the non‐AT group (74% vs 63%, 52% vs 27%, 42% vs 20%, all *P* < .05, respectively). Similarly, after 1:2 PSM, the median DFS time was still longer in the AT than in the non‐AT group (16.0 months vs 11.0 months, *P* = .046; Figure 2D), and the 1‐, 3‐, and 5‐year DFS rates were still significantly higher in the AT than in the non‐AT group (58% vs 45%, 35% vs 19%, 24% vs 13%, all *P* < .05, respectively).

### 3.3 Univariate and multivariate analyses of prognostic factors for OS and DFS in ICC patients following radical resection

A univariate analysis of the whole patient cohort identified gender, margin, tumor size, tumor number, satellite, LNM, and AT as prognostic factors for OS (all *P* < .05; Table 2). A subsequent multivariate analysis showed that being male (*P* = .004, HR: 1.48, 95% CI: 1.13‐1.93), a CA19‐9 >37 U/mL (*P* = .03, HR: 1.38, 95% CI: 1.03‐1.85), tumor size >5 cm (*P* = .002, HR: 2.27, 95% CI: 1.37‐3.77), satellite (*P* = .013, HR: 1.86, 95% CI: 1.14‐3.03), and LNM (*P* = .002, HR: 1.82, 95% CI: 1.25‐2.63) were all independent risk factors for OS (Table 2).

A univariate analysis of variables for DFS identified gender, margin, tumor size, tumor number, satellite, LNM, and AT as prognostic factors (all *P* < .05; Table 2). A multivariate analysis showed that gender (*P* = .004, HR: 1.42, 95% CI: 1.12‐1.79), tumor size (*P* = .006, HR: 1.80, 95% CI: 1.18‐2.74), satellite (*P* = .026, HR: 1.65, 95% CI: 1.06‐2.65), and LNM (*P* = .005, HR: 1.61, 95% CI: 1.15‐2.25) were all independent risk factors for DFS (Table 2).

### 3.4 Subgroup analysis stratified by risk factors

**Figure 3:**
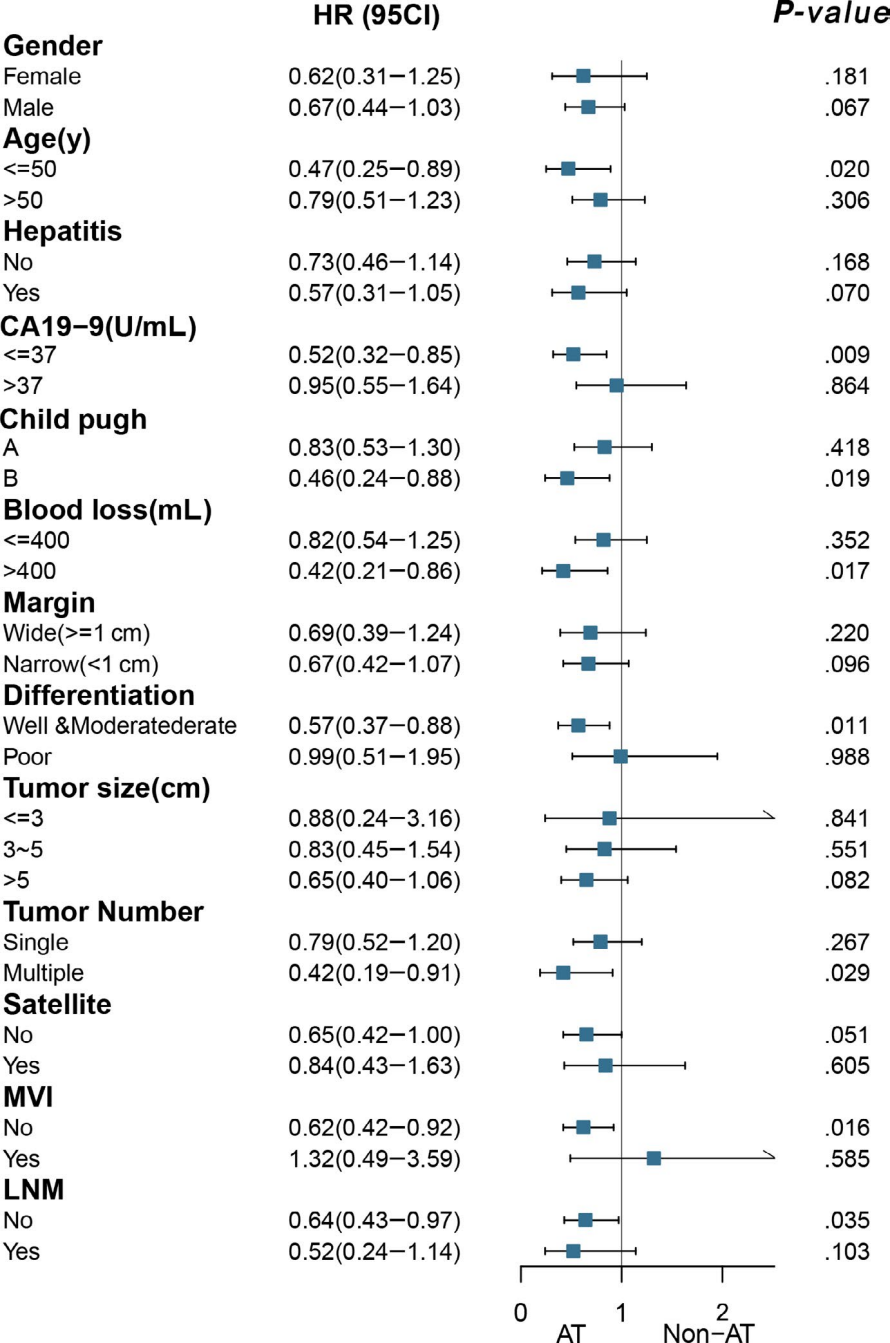
Forest plot of subgroup analysis stratified by risk factors

Subgroup analysis showed that patients with the following characteristics benefited from AT in terms of OS: age ≤50 years, CA19‐9 ≤37 U/mL, Child‐Pugh grading B, intraoperative blood loss >400 mL, well‐moderate tumor differentiation, and multiple tumors or no MVI (HR: 0.47, 95% CI: 0.25‐0.89, *P* = .02; HR = 0.52, 95% CI: 0.32‐0.85, *P* = .009; HR = 0.46, 95% CI: 0.24‐0.88, *P* = .019; HR = 0.42, 95% CI: 0.21‐0.86, *P* = .017; HR = 0.57, 95% CI: 0.37‐0.88, *P* = .011; HR = 0.42, 95% CI: 0.19‐0.91, *P* = .029; and HR = 0.62, 95% CI: 0.42‐0.92, *P* = .016, respectively; Figure 3).

### 3.5 Subgroup analysis based on established staging systems

**Figure 4:**
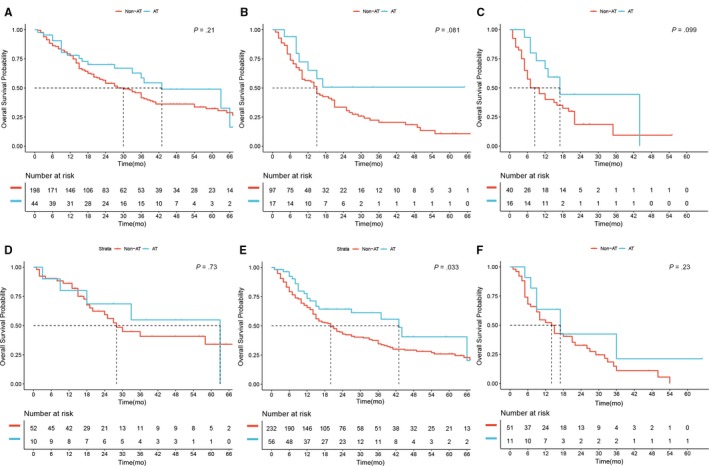
Overall survival (OS) of patients with stage Ⅰ (A), stage Ⅱ (B), and stage Ⅲ (C) according to the 8th AJCC stage system receiving adjuvant therapy (AT) or not. OS of patients with “low‐risk” (D), “middle‐risk” (E), and “high‐risk” (F) according to the established nomogram

Patients included in the study were divided into three subgroups according to the 8th AJCC staging system.^5^ In total, 242 (58.74%), 114 (27.67%), and 56 (13.59%) patients were in the stage Ⅰ, stage Ⅱ, and stage Ⅲ subgroups, respectively. Good prognostic stratification was observed among the three subgroups (*P* < .05; Figure S1A). However, the use of AT failed to improve the OS for patients at stage Ⅰ, Ⅱ, or Ⅲ (43.0 months vs 30.0 months, *P* = .21, Figure 4A; NA vs 15.0 months, *P* = .081, Figure 4B; 17.0 months vs 8.0 months, *P* = .099, Figure 4C, respectively).

According to the established nomogram,^11^ patients were divided into three risk subgroups. In total, 62 (15%), 288 (70%), and 62 (15%) patients were grouped into the “low‐risk,” “middle‐risk,” and “high‐risk” subgroups, all of which exhibited good prognostic stratification (*P* < .05; Figure S1B). The use of AT failed to improve OS for either “low‐risk” or “high‐risk” patients (63.0 months vs 28.0 months, *P* = .73, Figure 4D; 17.0 months vs 14.0 months, *P* = .23, Figure 4F, respectively). However, patients in the “middle‐risk” subgroup who received AT had a longer median OS (43.0 months vs 20.0 months, *P* = .033; Figure 4E).

### 3.6 Subgroup analysis stratified by different AT strategies

**Figure 5:**
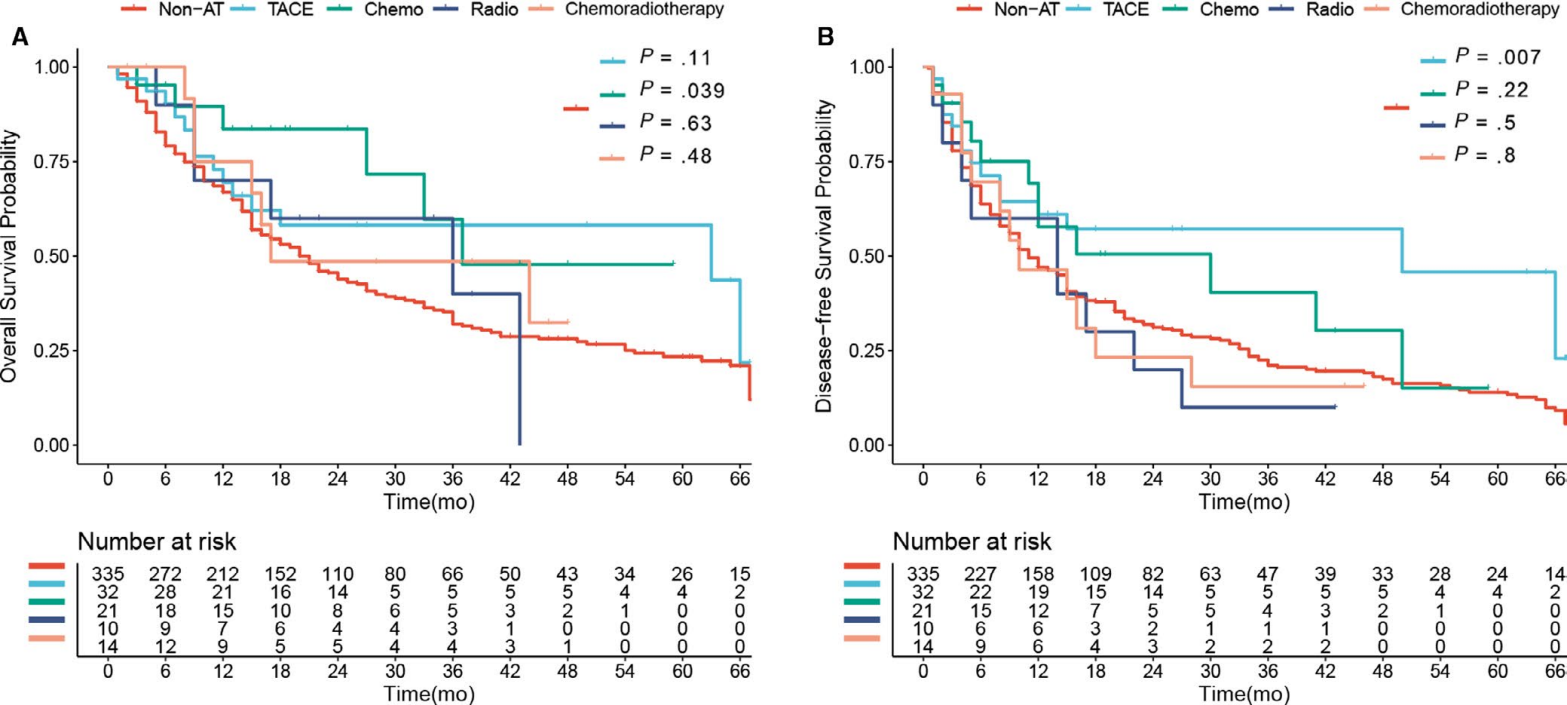
Overall survival (OS) (A) and disease‐free survival (DFS) (B) of patients receiving adjuvant TACE, chemotherapy, radiotherapy, and chemoradiotherapy, compared with those without adjuvant therapy

In total, 77 patients (18.9%) received AT after surgery before recurrence, including 32 (7.8%) patients who received adjuvant TACE, 21 (5.1%) patients who received adjuvant chemotherapy, 10 (2.4%) patients who received adjuvant radiotherapy, and 14 (3.4%) patients who received adjuvant chemoradiotherapy. Compared to the OS of non‐AT patients, the median OS in the adjuvant TACE, chemotherapy, radiotherapy, and chemoradiotherapy groups was as follows: 63.0 months vs 21.0 months (*P* = .11; Figure 5A), 37.0 months vs 21.0 months (*P* = .039; Figure 5A), 36.0 months vs 21.0 months (*P* = .63; Figure 5A), and 17.0 months vs 21.0 months (*P* = .48; Figure 5A), respectively. While the corresponding median DFS rates for each group were 8.0 months vs 10.0 months (*P* = .007; Figure 5B), 12.0 months vs 10.0 months (*P* = .22; Figure 5B), 4.0 months vs 10.0 months (*P* = .50; Figure 5B), and 5.0 months vs 10.0 months (*P* = .80; Figure 5B), respectively.

## 4 DISCUSSION

Various AT strategies have been used in ICC patients following radical resection to improve their prognosis; however, the benefit of AT has always been questioned.^18^, ^19^, ^20^, ^21^, ^22^, ^23^ In this study, we collected multicenter ICC patient data, including 77 patients who received AT after a radical resection, and the results showed that these patients had a longer median OS and DFS than who did not receive AT. These results were also confirmed after 1:2 PSM. Hence, AT should be recommended for patients with ICC, following radical resection. However, this did not indicate whether one group of ICC patients was more likely to benefit from AT than another, or which AT strategy was the most effective.

In a real‐world scenario, “high‐risk” patients are much more likely to receive AT following resection; however, the efficacy of AT varies among patients. When evaluating any clinical strategy, survival is the most convincing indicator of efficacy. Therefore, we performed risk factor subgroup analyses to determine the characteristics of ICC patients most likely to benefit from AT in terms of OS. The results showed that patients with the following characteristics could significantly benefit from undergoing AT: age ≤50 years, CA19‐9 ≤37 U/mL, Child‐Pugh grading B, intraoperative blood loss >400 mL, well‐moderate differentiation, multiple tumors, no satellite, or no MVI (all *P* < .05). However, this list of characteristics is confusing since being aged ≤50 years, having a CA19‐9 level ≤37 U/mL, well‐moderate tumor differentiation, and the presence of no satellites and MVI are often considered to be “protective factors” of OS, while a Child‐Pugh grading B and the presence of multiple tumors are “risk factors.” Hence, it is imperative to identify an appropriate staging system for the successful postoperative management of ICC patients.

The AJCC cancer staging system is the most widely used staging system to predict the prognosis of ICC worldwide. In this study, patients were divided into three groups according to the 8th AJCC cancer staging system for ICC, each with distinct OS differences^5^ (*P* < .05). However, this failed to confirm the benefit of AT in any of the subgroups. It has been reported that a prognostic nomogram based on prognostic factors, with corresponding weights based on their survival impact, is better than conventional staging systems for predicting patient prognosis.^11^ Therefore, we also divided patients into “low‐risk,” “middle‐risk,” and “high‐risk” subgroups, each with distinctly different survival rates (*P* < .05). We found that only “middle‐risk” patients benefited from receiving AT. This indicates that the established nomogram is superior to the AJCC cancer staging system in the postoperative management of ICC and that “middle‐risk” patients should be strongly recommended to receive AT following radical resection.

The incidence of ICC patients receiving adjuvant chemotherapy is reported to be as high as 46.6%,^10^ although cholangiocarcinoma is less sensitive to chemotherapeutics than other cancers.^18^, ^24^ Fluoropyrimidine‐ and gemcitabine‐based chemotherapy regimens are widely used^20^, ^25^ and were confirmed to prolong OS in recent meta‐analyses.^16^, ^17^ In this study, we also found that adjuvant chemotherapy could prolong the median OS, which is in accordance with the findings of previous reports.^19^, ^20^

TACE is often used to manage ICC in unresectable patients,^26^, ^27^ but more recently, adjuvant TACE has also been administered to reduce ICC recurrence.^7^, ^21^, ^23^ Previous studies have found that adjuvant TACE can improve ICC patient prognosis following resection, mainly by reducing the rate of recurrence. However, this has been questioned by Shen et al.^28^ In our opinion, adjuvant TACE might reduce the risk of recurrence when cancerous cells are carried in the blood, but it is insufficient in cases where they are spread through the lymph system.^29^, ^30^ In this study, we found that adjuvant TACE prolonged the median DFS but failed to prolong the median OS, which is consistent with our hypothesis. In future, an adjuvant combination therapy of chemotherapy and TACE should be trialed in a clinical setting.

Radiotherapy plays an increasingly important role in the management of ICC.^31^, ^32^ However, adjuvant radiotherapy has been found to be inefficient in improving the prognosis of ICC patients following R0 resection,^31^, ^32^ which was confirmed by this study. Data from the Taiwan Cancer Registry database found that more patients (53.4%) received adjuvant chemoradiotherapy following resection than radiotherapy alone following resection, and that concurrent rather than sequential chemoradiotherapy improved survival in patients at an advanced stage or with a positive margin.^10^ In this study, only 14 (3.4%) patients received adjuvant chemoradiotherapy, most of whom underwent sequential chemoradiotherapy, and our results showed that adjuvant chemoradiotherapy improved neither the OS nor the DFS. However, more trials should be conducted to identify the efficacy of adjuvant chemoradiotherapy in cases of ICC, especially with the advent of stereotactic body radiotherapy.

However, there were several limitations to this study. Firstly, this was a retrospective study and, although we conducted PSM, both selection and recall bias were hard to avoid. Secondly, adverse events related to AT were not evaluated, although no cases of mortality were detected. Finally, only 77 patients (18.9%) underwent AT, and therefore, our subgroup analysis was of limited significance. Hence, the AT resulting in the best survival should be explored further.

## 5 CONCLUSION

With the current data, we conclude that AT should be recommended in the management of ICC following radical resection, especially for those “middle‐risk” patients, as evaluated by the established nomogram. However, prospective trials are needed to determine the most beneficial AT strategy.

## CONFLICT OF INTEREST

None declared.

## AUTHOR CONTRIBUTIONS

Lei Wang, Jianying Lou, Shuguo Zheng, Xinyu Bi, Jianming Wang, Wei Guo, Fuyu Li, Jian Wang, Yamin Zheng, Jingdong Li, and Shi Cheng offering the data; Lei Wang, Manjun Deng, and Qiao Ke acquisition of data, analysis, and interpretation of data; Lei Wang drafting the article; Yongyi Zeng and Weiping Zhou conception and design of the study, revising the article, critical revision, and final approval.

## Data Availability

All data included in this study are available upon request by contact with the corresponding author.

## References

[1] Bray F , Ferlay J , Soerjomataram I , Siegel RL , Torre LA , Jemal A . Global cancer statistics 2018: GLOBOCAN estimates of incidence and mortality worldwide for 36 cancers in 185 countries. CA Cancer J Clin. 2018;68(6):394‐424. 30207593 10.3322/caac.21492

[2] Bertuccio P , Malvezzi M , Carioli G , et al. Global trends in mortality from intrahepatic and extrahepatic cholangiocarcinoma. J Hepatol. 2019;71(1):104‐114. 30910538 10.1016/j.jhep.2019.03.013

[3] Si A , Li J , Xiang H , et al. Actual over 10‐year survival after liver resection for patients with intrahepatic cholangiocarcinoma. Oncotarget. 2017;8(27):44521‐44532. 28562348 10.18632/oncotarget.17815 PMC5546499

[4] Zhang Y , Shi S‐M , Yang H , et al. Systemic inflammation score predicts survival in patients with intrahepatic cholangiocarcinoma undergoing curative resection. J Cancer. 2019;10(2):494‐503. 30719145 10.7150/jca.26890 PMC6360305

[5] Benson AB , D'Angelica MI , Abbott DE , et al. Guidelines Insights: Hepatobiliary Cancers, Version 2.2019. J Natl Compr Canc Netw. 2019;17(4):302‐310. 30959462 10.6004/jnccn.2019.0019

[6] Shen F , Xie ZH , Xia Y , Wu MC . Progress on surgical treatment of intrahepatic cholangiocarcinoma. Zhonghua Wai Ke Za Zhi. 2019;57(4):241‐246. 30929367 10.3760/cma.j.issn.0529-5815.2019.04.001

[7] Jeong S , Zheng BO , Wang J , et al. Transarterial chemoembolization: A favorable postoperative management to improve prognosis of hepatitis B virus‐associated intrahepatic cholangiocarcinoma after surgical resection. Int J Biol Sci. 2017;13(10):1234‐1241. 29104490 10.7150/ijbs.21149 PMC5666522

[8] Reames BN , Bagante F , Ejaz A , et al. Impact of adjuvant chemotherapy on survival in patients with intrahepatic cholangiocarcinoma: a multi‐institutional analysis. HPB. 2017;19(10):901‐909. 28728891 10.1016/j.hpb.2017.06.008

[9] Zheng X , Chen B , Wu JX , et al. Benefit of adjuvant radiotherapy following narrow‐margin hepatectomy in patients with intrahepatic cholangiocarcinoma that adhere to major vessels. Cancer Manag Res. 2018;10:3973‐3981. 30310318 10.2147/CMAR.S172940 PMC6165777

[10] Lin Y‐K , Hsieh M‐C , Wang W‐W , et al. Outcomes of adjuvant treatments for resectable intrahepatic cholangiocarcinoma: Chemotherapy alone, sequential chemoradiotherapy, or concurrent chemoradiotherapy. Radiother Oncol. 2018;128(3):575‐583. 29801723 10.1016/j.radonc.2018.05.011

[11] Li J , Wang Q , Lei Z , et al. Adjuvant transarterial chemoembolization following liver resection for intrahepatic cholangiocarcinoma based on survival risk stratification. Oncologist. 2015;20(6):640‐647. 25956404 10.1634/theoncologist.2014-0470 PMC4571785

[12] Miura JT , Johnston FM , Tsai S , et al. Chemotherapy for surgically resected intrahepatic cholangiocarcinoma. Ann Surg Oncol. 2015;22(11):3716‐3723. 25777092 10.1245/s10434-015-4501-8

[13] Jiang W , Zeng Z‐C , Tang Z‐Y , et al. Benefit of radiotherapy for 90 patients with resected intrahepatic cholangiocarcinoma and concurrent lymph node metastases. J Cancer Res Clin Oncol. 2010;136(9):1323‐1331. 20130909 10.1007/s00432-010-0783-1 PMC11828251

[14] Hammad AY , Berger NG , Eastwood D , et al. Is radiotherapy warranted following intrahepatic cholangiocarcinoma resection? The impact of surgical margins and lymph node status on survival. Ann Surg Oncol. 2016;23(Suppl 5):912‐920. 27654107 10.1245/s10434-016-5560-1

[15] Lee GC , Ferrone CR , Tanabe KK , et al. Predictors of adjuvant treatment and survival in patients with intrahepatic cholangiocarcinoma who undergo resection. Am J Surg. 2019;218(5):959‐966. 30871788 10.1016/j.amjsurg.2019.02.036 PMC6722029

[16] Ma KW , Cheung TT , Leung B , et al. Adjuvant chemotherapy improves oncological outcomes of resectable intrahepatic cholangiocarcinoma: A meta‐analysis. Medicine. 2019;98(5):e14013. 30702559 10.1097/MD.0000000000014013 PMC6380775

[17] Wang ML , Ke ZY , Yin S , Liu CH , Huang Q . The effect of adjuvant chemotherapy in resectable cholangiocarcinoma: A meta‐analysis and systematic review. Hepatobiliary Pancreat Dis Int. 2019;18(2):110‐116. 30470543 10.1016/j.hbpd.2018.11.001

[18] Shroff RT , Kennedy EB , Bachini M , et al. Adjuvant therapy for resected biliary tract cancer: ASCO Clinical Practice Guideline. J Clin Oncol. 2019;37(12):1015‐1027. 30856044 10.1200/JCO.18.02178

[19] Tran CH , Zhang Q , Sada YH , Chai C , Curley SA , Massarweh NN . The role of surgery and adjuvant therapy in lymph node‐positive cancers of the gallbladder and intrahepatic bile ducts. Cancer. 2018;124(1):74‐83. 28841223 10.1002/cncr.30968

[20] Schweitzer N , Weber T , Kirstein MM , et al. The effect of adjuvant chemotherapy in patients with intrahepatic cholangiocarcinoma: a matched pair analysis. J Cancer Res Clin Oncol. 2017;143(7):1347‐1355. 28314929 10.1007/s00432-017-2392-8 PMC11819035

[21] Li T , Qin L‐X , Zhou J , et al. Staging, prognostic factors and adjuvant therapy of intrahepatic cholangiocarcinoma after curative resection. Liver Int. 2014;34(6):953‐960. 24134199 10.1111/liv.12364

[22] Liu RQ , Shen SJ , Hu XF , Liu J , Chen LJ , Li XY . Prognosis of the intrahepatic cholangiocarcinoma after resection: hepatitis B virus infection and adjuvant chemotherapy are favorable prognosis factors. Cancer Cell Int. 2013;13(1):99. 24139471 10.1186/1475-2867-13-99 PMC3852727

[23] Wu ZF , Zhang HB , Yang N , Zhao WC , Fu Y , Yang GS . Postoperative adjuvant transcatheter arterial chemoembolisation improves survival of intrahepatic cholangiocarcinoma patients with poor prognostic factors: results of a large monocentric series. Eur J Surg Oncol. 2012;38(7):602‐610. 22417704 10.1016/j.ejso.2012.02.185

[24] Messina C , Merz V , Frisinghelli M , et al. Adjuvant chemotherapy in resected bile duct cancer: A systematic review and meta‐analysis of randomized trials. Crit Rev Oncol Hematol. 2019;143:124‐129. 31563828 10.1016/j.critrevonc.2019.09.002

[25] Morine Y , Shimada M , Ikemoto T , et al. Effect of adjuvant gemcitabine combined with low‐dose 5‐fluorouracil and cisplatin chemotherapy for advanced biliary carcinoma. Anticancer Res. 2017;37(11):6421‐6428. 29061828 10.21873/anticanres.12096

[26] Hyder O , Marsh JW , Salem R , et al. Intra‐arterial therapy for advanced intrahepatic cholangiocarcinoma: a multi‐institutional analysis. Ann Surg Oncol. 2013;20(12):3779‐3786. 23846786 10.1245/s10434-013-3127-y

[27] Park SY , Kim JH , Yoon HJ , Lee IS , Yoon HK , Kim KP . Transarterial chemoembolization versus supportive therapy in the palliative treatment of unresectable intrahepatic cholangiocarcinoma. Clin Radiol. 2011;66(4):322‐328. 21356394 10.1016/j.crad.2010.11.002

[28] Shen WF , Zhong W , Liu Q , Sui CJ , Huang YQ , Yang JM . Adjuvant transcatheter arterial chemoembolization for intrahepatic cholangiocarcinoma after curative surgery: retrospective control study. World J Surg. 2011;35(9):2083‐2091. 21698503 10.1007/s00268-011-1171-y

[29] Zhou R , Lu D , Li W , et al. Is lymph node dissection necessary for resectable intrahepatic cholangiocarcinoma? A systematic review and meta‐analysis. HPB. 2019;21(7):784‐792. 30878490 10.1016/j.hpb.2018.12.011

[30] Hu J , Chen F‐Y , Zhou K‐Q , et al. Intrahepatic cholangiocarcinoma patients without indications of lymph node metastasis not benefit from lymph node dissection. Oncotarget. 2017;8(69):113817‐113827. 29371948 10.18632/oncotarget.22852 PMC5768365

[31] Kasuya G , Terashima K , Shibuya K , et al. Carbon‐ion radiotherapy for cholangiocarcinoma: a multi‐institutional study by and the Japan carbon‐ion radiation oncology study group (J‐CROS). Oncotarget. 2019;10(43):4369‐4379. 31320991 10.18632/oncotarget.27028 PMC6633891

[32] Brunner TB , Blanck O , Lewitzki V , et al. Stereotactic body radiotherapy dose and its impact on local control and overall survival of patients for locally advanced intrahepatic and extrahepatic cholangiocarcinoma. Radiother Oncol. 2019;132:42‐47. 30825968 10.1016/j.radonc.2018.11.015

