# Supplementary figures and images for: Postoperative adjuvant therapy following radical resection for intrahepatic cholangiocarcinoma: A multicenter retrospective study
Source: Cancer Med. 2020 Feb 19;9(8):2674–85. doi: 10.1002/cam4.2925 (PMC7163087; doi:10.1002/cam4.2925)

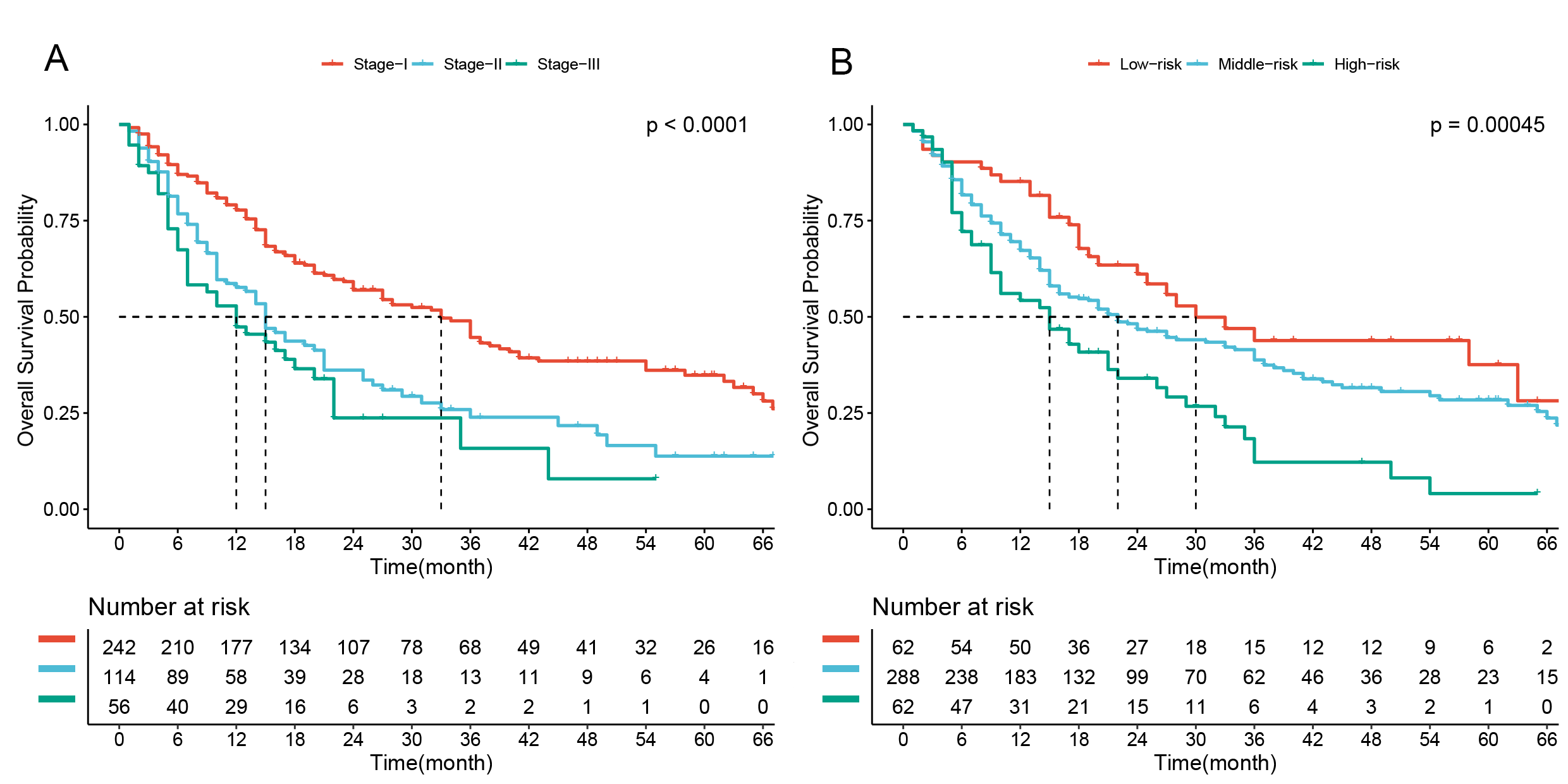

Supplement: Supplementary file 1 [file CAM4-9-2674-s001.tif]
